# Abdominopelvic CT-scan in emergency departments for patients with suspected complications of Crohn’s disease: a single tertiary center experience

**DOI:** 10.1186/s12873-021-00512-5

**Published:** 2021-10-07

**Authors:** Mikael Verdalle-Cazes, Cloé Charpentier, Coralie Benard, Luc-Marie Joly, Jean-Nicolas Dacher, Guillaume Savoye, Céline Savoye-Collet

**Affiliations:** 1 Department of Radiology, Normandie University, UNIROUEN, Quantif-LITIS EA 4108, Rouen University Hospital-Charles Nicolle, 1 rue de Germont, F-76031 Rouen Cedex, France; 2 Department of Gastroenterology, Rouen University Hospital-Charles Nicolle, 1 rue de Germont, F-76031 Rouen Cedex, France; 3 Department of Emergency, Rouen University Hospital-Charles Nicolle, 1 rue de Germont, F-76031 Rouen Cedex, France; 4 Department of Radiology, Rouen University Hospital-Charles Nicolle, 1 rue de Germont, F-76031 Rouen Cedex, France; 5 Department of Gastroenterology, Normandie University, UNIROUEN, INSERM U1073, Rouen University Hospital-Charles Nicolle, 1 rue de Germont, F-76031 Rouen Cedex, France

**Keywords:** Crohn’s disease, Emergency department, Abdominopelvic CT-scan

## Abstract

**Background:**

Crohn’s disease (CD) is a chronic disorder with frequent complications. The objective of this study was to assess the predictive factors of finding a complication of CD using abdominopelvic CT-scan in patients with a visit to the emergency department.

**Methods:**

Patients with at least one visit to the gastroenterology department of our University hospital during the year with a CD were retrospectively included. All visits to the emergency department of the hospital during the follow-up of these patients were identified.

**Results:**

A total of 638 patients were included and 318 (49.8%) had at least one visit to the emergency department since the beginning of their follow-up. Abdominopelvic CT-scan was performed in 141 (23.7%) of the 595 visits for digestive symptoms. Only 4.3% of these CT-scans were considered as normal; there was luminal inflammation without complication in 24.8%, abscess, fistula or perforation in 22.7%, mechanical bowel obstruction in 36.9% and diagnosis unrelated to CD in 11.3%. In univariate analysis, stricturing phenotype (OR, 2.48; 95% CI, 1.16–5.29; *p* = 0.02) and previous surgery (OR, 2.90; 95% CI, 1.37–6.14; *p* = 0.005) were predictive factors of finding a complication of CD using abdominopelvic CT-scan, whereas no independent predictive factor was statistically significant in multivariate analysis.

**Conclusion:**

In CD patients consulting in emergency department, CT-scan examination was performed in 24% of visits for digestive symptoms and complications of CD were found in 60%. Complications were more frequent in patients with stricturing phenotype and previous surgery.

## Introduction

### Background

Crohn’s disease (CD) is a chronic, transmural, immune-mediated disorder that affects the gastrointestinal tract [1]. The main clinical symptoms are abdominal pain, fever and clinical signs of bowel obstruction or diarrhea. Any section of the digestive tract can be affected and extraintestinal manifestations are possibly associated. CD is more frequently diagnosed in young people in the second to fourth decade of life. The prevalence of CD is 3.2 per 1000 people in Europe [2].

CD is a chronic disease whose management is mainly ambulatory. However, the evolution of CD is difficult to predict with a rather high risk of complications needing an hospitalization, often after an admission to the emergency department [3]. Emergency room visits for inflammatory bowel diseases in the United States increased by 165% between 1994 and 2005 [4]. It has been proposed that this increase could be due to a rise of the incidence of CD, a higher severity of the disease and a delayed use of medical care [4]. More generally, the use of emergency services significantly increased since the 2000s in the general population, particularly in patients with chronic conditions [5, 6].

When patients with CD are admitted to emergency department for digestive symptoms, 50 to 70% of them benefit from abdominopelvic CT-scan [3, 7–9]. The number of CT-scans performed in emergency departments almost doubled for these patients since the 2000s [3]. Because CD is a chronic disease, irradiation is a concern and should be limited during the follow-up. Nevertheless, the diagnosis of acute stricturing or penetrating complications remains an indication for abdominopelvic CT-scan [10].

The profile of CD patients who visit emergency department and the characteristic of their disease are poorly known. The objective of this study was to assess the predictive factors of finding a complication of CD with abdominopelvic CT-scan in patients with a visit to the emergency department.

## Materials and methods

### Study design

This retrospective study was performed in a unique center (University hospital of Rouen) in a cohort of patients with CD. This study was performed in accordance with relevant guidelines and regulations. According to French law, written informed consent was waived (Rouen University Institutional Review Board - n°E2019–65).

### Selection of patients

Patients with at least one visit to the gastroenterology department of the University hospital of Rouen in 2014 were screened with the code K50.9 (Crohn’s disease, unspecified) of the International Classification of Diseases 10th edition (ICD-10). Patients with an uncertain diagnosis of CD or undefined colitis were excluded. All visits to the emergency department of the hospital since the beginning of the follow-up in the gastroenterology department were identified using the hospitalization database of the hospital.

### Data collection

Patient data obtained retrospectively from electronic medical files included: age, gender, smoking status, date of diagnosis of CD, Montreal classification at diagnosis, duration of follow-up in the gastroenterology department, change of phenotype during the follow-up, extraintestinal manifestations of CD (musculoskeletal and cutaneous), previous treatments of CD (5-aminosalicylic acid, systemic or topical corticosteroids, immunosuppressants, anti-TNF therapy, artificial nutrition), duration of anti-TNF therapy, surgery, number of unscheduled hospital stays (gastroenterology department or digestive surgery department) and number of outpatient visits to the gastroenterology department. If missing in electronic medical record, data were searched in patient’s paper file.

For each included patient, the number of admissions to the emergency department of the hospital was assessed from the beginning of the follow-up in the gastroenterology department. For patients with at least one admission to the emergency department, additional data were recorded: disease characteristics at the admission in emergency department (phenotype of the disease, on-going treatment, change or optimization of treatment within last 3 months, surgery within last 3 months), data on admission in the emergency department (date, main reason for admission, CT-scan), biological parameters (C-reactive protein), management of patient after visit to the emergency department (hospitalization in gastroenterology department or digestive surgery department, consultation in gastroenterology department within 3 months, change or optimization of treatment within 3 months, medical surgery within 3 months).

### Statistical analysis

Qualitative data were analyzed with Chi-square test and quantitative data with ANOVA test. A multivariate regression analysis was performed to assess the predictive factors of finding a complication of CD using abdominopelvic CT-scan. Only variates with less than 10% of missing data were included in the univariate analysis. Variates with *p* < 0.1 in univariate analysis were selected and included in the multivariate analysis.

Tests were two-sided and a *p*-value lower than 0.05 was considered to be statistically significant. SPSS software was used for statistical analyses (SPSS Inc., Chicago, IL, USA).

## Results

### General characteristics of patients

**Fig. 1:**
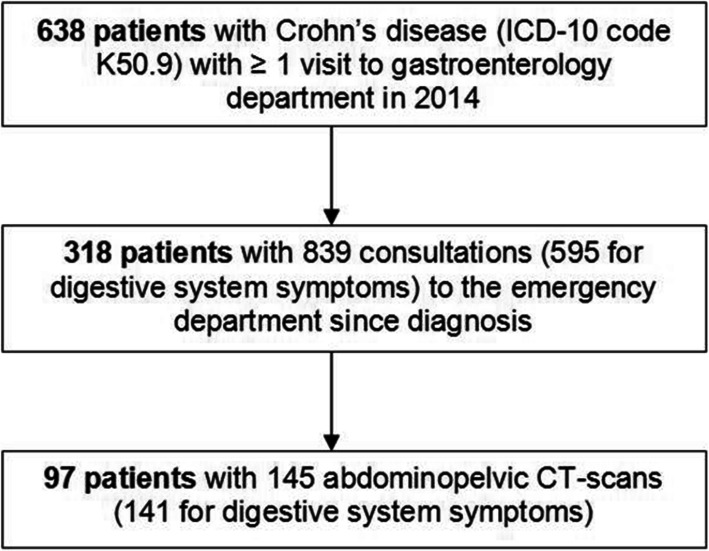
Flow chart

During 1 year, 638 patients with CD had at least one consultation in the gastroenterology department in 2014 (Fig. 1). Women were 40.1% and mean (SD) age at diagnosis was 27.3 (12.1) years (with 75.1% of patients between 17 and 40 years) (Table 1). The mean (SD) disease duration was 146 (118) months. The most frequent phenotype was inflammatory (80.2%) and the most frequent localization was ileocolonic (43.1%). Extraintestinal manifestations were reported in 27% (*n* = 174) of patients (musculoskeletal, 16.0%; cutaneous, 6.1%; both, 5.2%).

**Table 1:** Characteristics of patients with Crohn’s disease with at least one visit to the gastroenterology department in 2014

Characteristics	***N*** = 638
Women, n (%)	256 (40.1)
Age (years) at diagnosis	
Mean (SD)	27.3 (12.1)
Age classes, n (%)	
< 16	82 (12.9)
17–40	479 (75.1)
> 40	77 (12.1)
Phenotype at diagnosis, n (%)	
Inflammatory	512 (80.2)
Stenosing	35 (5.5)
Penetrating	91 (14.3)
Localization of disease at diagnosis, n (%)	
L1	199 (31.2)
L2	160 (25.1)
L3	275 (43.1)
L4	4 (0.6)
Active smokers, n (%) ^a^	221 (53.0)
Duration (months) of disease, mean (SD)	146 (118)
Extraintestinal manifestations, n (%)	174 (27.3)
Musculoskeletal	102 (16.0)
Cutaneous	39 (6.1)
Both	33 (5.2)

^a^ For patients with available data

L1, terminal ileum; L2, colon; L3, ileocolon; L4, upper gastrointestinal tract

### Indications for abdominopelvic CT-scan in emergency department and characteristics of patients at admission

Among the 638 patients, 318 (49.8%) consulted at the emergency department at least once since the diagnosis of CD for a total of 839 consultations. The average length of follow-up for the CD was 171 months (i.e., 14.3 years). The number of visits to the emergency department was 1–2 for 64% of patients (*n* = 205) and ≥ 3 for 36% (*n* = 113).

**Fig. 2:**
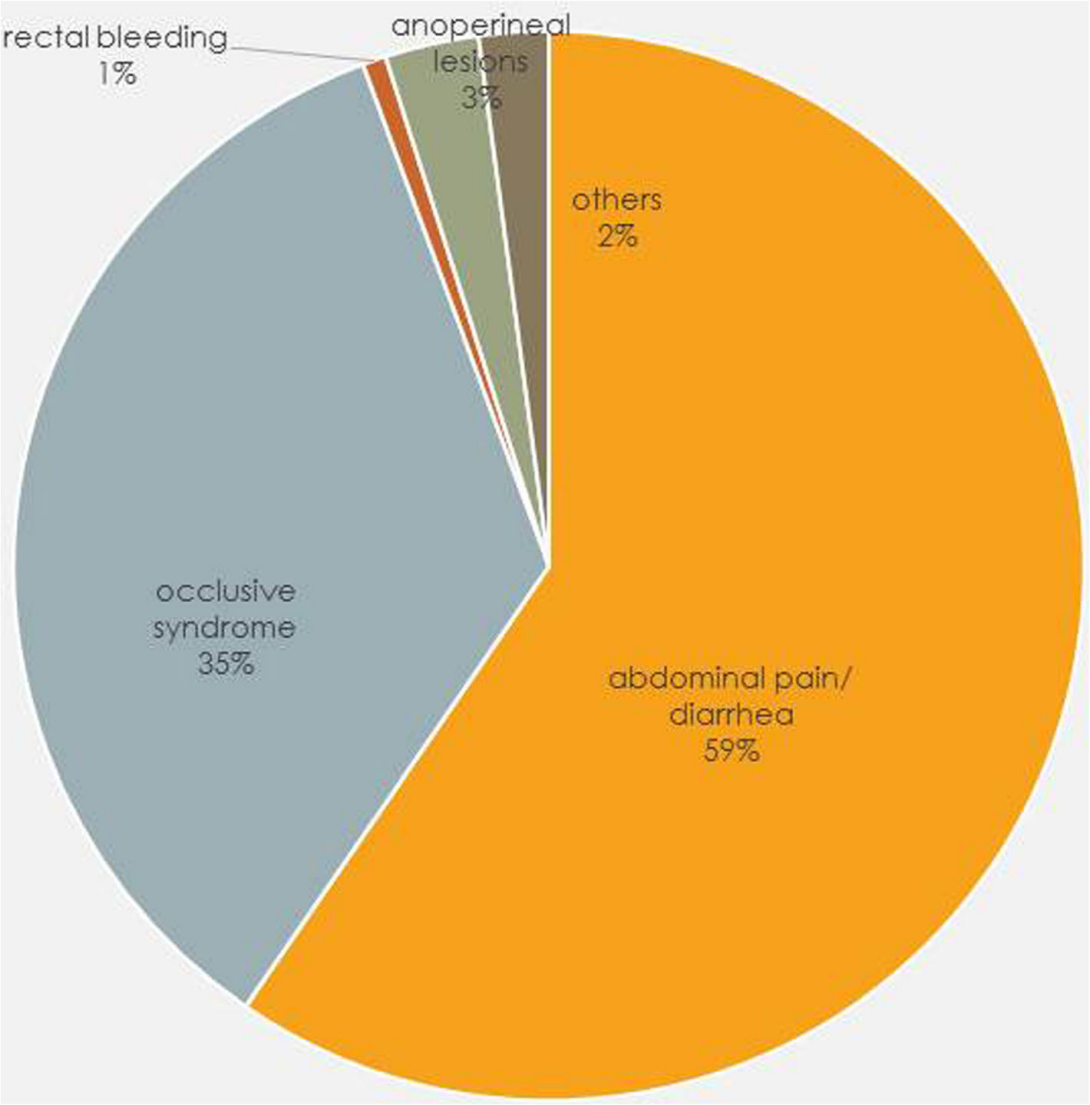
Reasons for emergency consultation in patients suffering from digestive symptoms

The main reasons for consultation were digestive symptoms (diarrhea, abdominal pain, rectal bleeding, occlusive syndrome, anoperineal lesions) that accounted for 70.9% of all consultations (Table 2 and Fig. 2). There were 2.4% of consultations due to extraintestinal manifestations of Crohn.

**Table 2:** Reasons for consultation in emergency department

Reasons for consultation	***N*** = 839
Digestive symptoms	595 (70.9)
Fever	38 (4.5)
Extraintestinal manifestations	20 (2.4)
Poor medical condition	14 (1.7)
Psychiatric symptoms	33 (3.9)
Cardio-pulmonary symptoms	35 (4.2)
Others	89 (10.6)
Missing	15 (1.8)

Results are given as n (%)

Among patient’s visits to emergency department, disease was inflammatory at admission in 34.4% of cases at admission, stricturing in 30.3% and penetrating in 35.3%. The pharmacological treatments at admission were immunosuppressants (22.6%; *n* = 190), 5-aminosalicylic acid alone (22.4%; *n* = 188), anti-TNF therapy (20.7%; n = 174), corticosteroids alone (7.7%; n = 65), combination of anti-TNF therapy and immunosuppressant (7.7%; *n* = 65) and artificial nutrition alone (1.1%; n = 8). For 149 visits (17.8%), patient had no treatment at admission. Treatment had been changed or optimized within 3 months before admission in 20.2% of cases and surgery had been performed within 3 months in 6.5%.

A total of 145 abdominopelvic CT-scans was performed in 97 patients. Eighty-two (56.6%) abdominopelvic CT-scans were done in men and 63 (43.4%) in women. The mean (SD) age at CT-scan was 42.0 (18.7) years. Out of the 595 admissions for digestive system symptoms, an abdominopelvic CT-scan was performed in 23.7% (*n* = 141) of cases. Biological tests (CRP, total blood count) were performed in 93.9% of visits.

### Abdominopelvic CT-scan reports

**Fig. 3:**
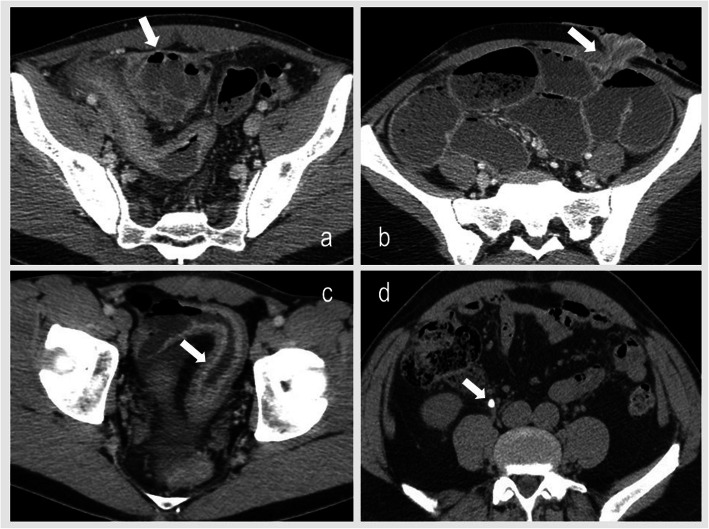
Examples of diagnosis made by CT scan: **a** abdominal abcess in a 36-years old patient (white arrow); **b** bowel obstruction related to a stenosis of the stomia in a 25-years old patient (white arrow); **c** ileal inflammation in a 21-years old patient (white arrow) and **d** right ureteral lithiasis in a 48-years old patient (renal colic)

Abdominopelvic CT-scan were realized with one or two acquisitions for a mean (SD) dose length product of 768 (565) mGy.cm. Only 4.3% (*n* = 6) of the 141 CT-scans were considered as normal (no detectable intra-abdominal acute event). There was luminal inflammation without complication in 24.8% (*n* = 35) of cases, abscess, fistula or perforation in 22.7% (*n* = 32), mechanical bowel obstruction in 36.9% (*n* = 52) and acute intra-abdominal diagnosis not related to CD in 11.3% (*n* = 16) (renal colic, acute cholecystitis) (Fig. 3).

### Predictive factors of finding a complication at CT-scan

The analysis of predictive factors of finding a complication of CD at abdominopelvic CT-scan is presented in Table 3. In univariate analysis, a stricturing phenotype (OR, 2.48; 95% CI, 1.16–5.29; *p* = 0.02) and a previous surgery (OR, 2.90; 95% CI, 1.37–6.14; *p* = 0.005) were predictive factors, whereas in multivariate analysis no independent predictive factor was statistically significant.

**Table 3:** Predictive factors of finding a complication of Crohn’s disease at abdominopelvic CT-scan in visits to the emergency department for digestive symptoms

	Complication CT-scan		Univariate analysis		Multivariate analysis
	No (*n* = 41)	Yes (*n* = 100)	*P*-value	Odds-ratio	*P*-value
**Biology**					
CRP (mean), mg/L	81	58	0.12		
**Phenotype at CT-scan**					
B1 (*n* = 44)	19 (46.3)	25 (25.0)		1 (ref)	
B2 (n = 44)	8 (19.5)	36 (36.0)	0.01	3.42 [1.30–9.03]	
B3 (*n* = 53)	14 (34.1)	39 (39.0)	0.11		
B2 or B3 (*n* = 97)	22 (53.6)	75 (75.0)	0.02	2.48 [1.16–5.29]	0.34
**Anamnesis**					
Recent treatment changes (< 3 months)	11 (26.8)	24 (24.0)	0.78		
Recent surgery (< 3 months)	1 (2.4)	10 (10.0)	0.12		
History of surgery	16 (39.0)	65 (65.0)	0.005	2.90 [1.37–6.14]	0.08

*CRP* C-reactive protein, *ref.* reference

B1, non-stricturing non-penetrating; B2, stricturing; B3, penetrating

### Diagnosis and management after visit to emergency department

After the admission to the emergency department, 96 patients were hospitalized in gastroenterology department or digestive surgery department. Medical treatment was changed or optimized in 45 and surgery was performed in 21 within 3 months.

## Discussion

In our large cohort of patients admitted to the emergency department of our hospital, CT-scan examination was performed in 24% of visits for digestive symptoms.

Patient characteristics are comparable to those reported in the literature for disease localization, phenotype and surgery [11–14]. The percentage of patients with a diagnosis between 17 and 40 years was high at 75.1% compared to literature where a percentage of 55% has been reported [13, 14]. However, the mean age at diagnosis of our cohort is comparable to other studies [14].

Our results show that CT-scan detected a penetrating (abscess, fistula, perforation) or stricturing complication (bowel obstruction) in 60% of cases, in contrast with the rates of complications reported in literature of only 23 to 36% [7–9, 15, 16].

Univariate analysis of our data evidenced that predictive factors of complications using CT-scan (perforation, abscess, fistula, stenosis) were history of abdominal surgery (OR, 2.9) and stricturing phenotype (OR, 3.42). No independent predictive factor was identified with multivariate analysis, may be due to our small sample size (141 CT-scans). In studies with higher sample sizes, independent predictive factors of abnormal findings using abdominopelvic CT-scan in CD patients presenting to an emergency department were history of abdominal surgery (OR, 2.2) [9], history of bowel obstruction (OR, 3.8), history of intraabdominal abscess (OR, 2.6) [17] and stricturing or penetrating phenotype (OR, 2.72) [16]. Nowadays, there is no available predictive score validated in independent population of CD’s patients for the diagnosis of complications. Clinical examination remains the predominant criteria for addressing the patient to the imaging unit.

The percentage of abdominopelvic CT-scans during visits for digestive system symptoms in patients with CD was 23.7% in our study. The study of Kerner et al. showed a significant increase of the rate of CT-scans performed in patients with CD admitted to emergency department: 47.1% in 2001 and 77.5% in 2009 [3]. A more frequent use of CT-scan was reported for all patients who were admitted to an emergency department for abdominal pain [18]. This is most probably due to the improved availability of CT-scans. Various studies reported rates from 49 to 71% for the use of CT-scan for abdominal pain in emergency departments [5, 7–9]. Therefore, the rates of CT-scan use that we report (23.7%) are relatively low, probably due to adequate patient selection in a tertiary center and 24-h availability of a gastroenterologist.

According to these results, the indication of CT-scan in emergency department for patients of our cohort appeared to be most often appropriate (low rates of CT-scans performed at admission and high rates of complications detected). Indeed, patients with CD are frequently irradiated for diagnosis purposes and it is necessary to limit their exposition to X-rays [19]. Recent studies in children showed that early exposure to radiations of CT-scan was associated to an increased risk of brain tumor and leukemia [20]. Several studies evidenced that CD patients received large cumulative doses (> 100 mSv) during their follow-up and were exposed to CT-scan radiations up to 2–3 times per year [19, 21–23].

According to the 2017 guidelines from the European Crohn’s and Colitis Organisation (ECCO), there is no indication for abdominopelvic CT-scan in suspected CD [10]. In this case, ileocolonoscopy and biopsies for microscopic evidence of CD are recommended as first-line procedure for the diagnosis. CT-scan, together with magnetic resonance imaging and trans-abdominal ultrasonography, are considered as complementary methods to endoscopy. Guidelines recommend to consider radiation exposure when selecting detection methods and especially for the follow-up [10]. The study of Kroeker et al. showed that 30% of the exposure to X-rays of patients with inflammatory bowel disease occurred during the admission to emergency unit including 75% with CT-scan [23].

Our low rate of CT in this clinical condition (CD’ patients consulting in emergency) could also have local explanation. All physicians in the emergency department could easily access to the total medical history of patients via our hospital computer network. Physicians in this department are well aware of the importance to limit X-Ray exposure in these patients. Hospitalization in a dedicated unit in gastroenterology is also a possibility for them, associated to a senior advice the next day and/or if required in a middle term an MR-enterography. Moreover, some patients went twice to the emergency department and CT could have been done at the second visit for persisting symptoms.

Education of patients on their condition could be a useful tool for limiting the number of admissions to emergency department and the exposition of CD patients to X-ray. Thus, the Spanish study of Casellas et al. reported that among patients with intestinal bowel disease that consulted in an emergency department, 20% of them considered that their visit could have been avoided if they had received a better information on their condition and 18% if they had disposed of the direct phone number of the gastroenterology department [24]. Only 37% considered that the information they received on their disease (evolutive potential, possible complications) was adequate. In CD, some studies showed that telephone follow-up led to a decrease of the number of hospitalizations and admission to emergency department [25].

The first limitation of our study was the limited sample size. This study has also some others limitations. Some of them are related to the retrospective design. For some parameters (e.g., smoking status, familial history), the rate of missing data was high and was a limitation for the analysis. This study was monocentric and was performed in a tertiary center. As a consequence, the cohort could not reflect all patients with CD. Some patients were not followed in our hospital at the onset of the disease. Therefore, visits to an emergency department during this early period could not be considered. However, the number of patients concerned is probably limited because most of severe CD or with complications were managed in our University Hospital. Biological data at the admission were not studied as predictive data because mechanical complication was also a potential diagnosis (37% of patients).

In conclusion, in CD patients consulting in emergency department, CT-scan examination was performed in 24% of visits for digestive symptoms and complications were found in 60%. Complications were more frequent in patients with stricturing phenotype and previous surgery. Clinical examination and medical history via hospital network remain important data for decision making in order to limit X-ray exposure.

## Data Availability

The datasets used and/or analysed during the current study are available from the corresponding author on reasonable request.
